# Design, Synthesis and antiHIV activity of Novel Isatine-Sulphonamides

**DOI:** 10.4103/0250-474X.49121

**Published:** 2008

**Authors:** P. Selvam, N. Murugesh, M. Chandramohan, Z. Debyser, M. Witvrouw

**Affiliations:** Arulmigu Kalasalingam College of Pharmacy, Anand nagar, Krishnankoil-626 190, India; 1 Institute of Pharmacology, Madurai Medical College, Madurai-625 020, India; 2 Bharat Ratna Kamarajar Liver Hospital and Research Centre, Madurai-625 001, India; 3 Molecular Medicine, Katholieke Universiteit Leuven and IRC KULAK, Leuven, Flanders, Belgium

**Keywords:** Isatin, HIV-1, MT-4 cells, HIV Integrase, sulphadimidine

## Abstract

A series of novel isatine-sulphonamide derivatives have been synthesized by combining isatin derivatives with sulphonamides. The structure of the synthesized compounds were elucidated by spectral analysis (IR, NMR and Mass). Investigation of anti-HIV activity was done against HIV-1(IIIB) in MT-4 cells and HIV integrase inhibitory activity. 4-(1-acetyl-5-methyl-2-oxoindolin-3-ylideneamino)-N-(4,6-dimethylpyrimidin-2-yl)benzenesulfonamide (SPIII-5ME-AC) inhibits the HIV Integrase enzymatic activity as both over all and strand transfer reaction and 4-(1-benzoyl-5-chloro-2-oxoindolin-3-ylideneamino)-N-(4,6-dimethylpyrimidin-2-yl)benzene sulfonamide (SPIII-5Cl-BZ) exhibits 36 percent maximum protection against HIV-1 at sub toxic concentration.

Isatin (2,3-dioxoindole), a versatile lead molecule for potential bioactive agents and its derivatives were reported to possess wide spectrum of activity. Methisazone (N-methylisatin-ß-thiosemicarbazone) was one of the first clinically used synthetic antiviral agents^1^. N-Methyl isatin-β-4':4'-diethylthiosemicarbazone was found to inhibit Maloney leukemia virus replication^2^. N,N-disubstituted thiosemicarbazone derivatives of isatin were tested for inhibition of HIV-1 replication^3^. Schiff and Mannich bases of isatin derivatives were synthesized and evaluated for antiviral activity. Some of their derivatives showed significant inhibitory activity against the replication of HIV-1^4^–^10^. In earlier studies, some novel isatin derivatives were synthesized and evaluated for antiviral, anticancer and antibacterial activities^11^,^12^. These compounds showed significant inhibitory effects against HIV-1 replication. In this study we describe the antiviral activity of some novel of isatine-sulphonamide derivatives (Scheme 1) against HIV-1 in MT-4 cells. These studies prompted us to investigate their inhibitory effect against HIV integrase.

**Scheme 1:**
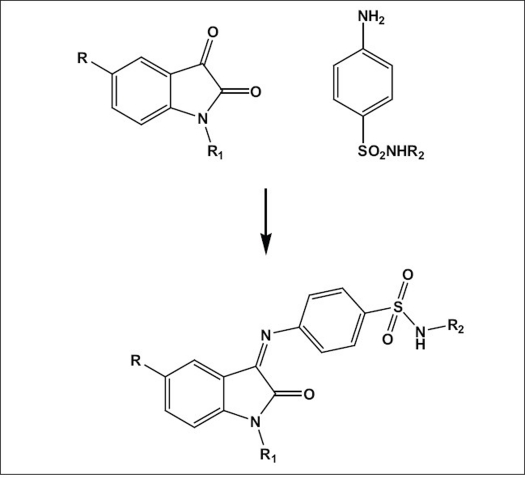
synthesis of isatinsulphonamide derivatives For SPIII-S, R is H, R_1_ is H and R_2_ is H; for SPIII-SMe, R is CH_3_, R_1_ is H and R_2_ is H; for SPIII-SCl, R is Cl, R_1_ is H and R_2_ is H; for SPIII-SM, R is Cl, R_1_ is H and R_2_ is 4,5-dimethyl-2-isoxazolyl; for SPIII-5Br-AC, R is Br, R_1_ is COCH_3_ and R_2_ is 4,6-dimethyl-2-pyrimidinyl; for SPIII-5Br-BZ, R is Br, R_1_ is COC_6_H_5_ and R_2_ is 4,6-dimethyl-2-pyrimidinyl; for SPIII-5Me-AC, R is CH_3_, R_1_ is COCH_3_ and R_2_ is 4,6-dimethyl-2-pyrimidinyl and for SPIII-5Cl-BZ, R is Cl, R_1_ is COC_6_H_5_ and R_2_ is 4,6-dimethyl-2-pyrimidinyl

Melting points were determined using Thomas melting point apparatus and are uncorrected. The purity was checked by TLC using silica gel G as stationary phase. The structure of the synthesized compounds was elucidated using a Perkin Elmer FT-IR in KBr disc and PMR was taken on a Bruker AMX-(400 MHz) FT-NMR. Mass spectra were obtained on a Varian Atlas CH-7 Mass spectrometer at 70 eV.

Isatine-sulphonamide derivatives were synthesized by refluxing an equimolar (0.01 mol) mixture of isatins (isatin, 5-chloro and 5-methyl) and sulphonamides (sulphonilamide and sulphamoxazole) for 6 h in 10 ml of glacial acetic acid. The mixture was cooled to room temperature and poured into crushed ice; the solid thus obtained was recrystallized from ethanol. SPIII-S yield: 68%, mp: 116°, IR (KBr) cm^−1^: 3300 (NH), 1510 (C=N), 1674 (C=0), 1583 (C=C), PMR (DMSO-d_6_) δ ppm: 2 (b, 2H, NH_2_), 7.1-7.9 (m, 8H, Ar-H), 8.1 (s, 1H, NH), EI- MS (m/e): 301.32. SPIII-SMe yield: 72%, mp: 128°, IR (KBr) cm^−1^: 3320 (NH), 1590 (C=N), 1650 (C=0), 1522 (C=C), PMR (DMSO-d_6_) δ ppm: 2.35 (s, 3H, CH_3_), 2.1 (b, 2H, NH_2_), 7.0-7.9 (m, 7H, Ar-H), 8.2 (s, 1H, NH), EI-MS (m/e): 315.35. SPIII-SCl yield: 82%, mp: 181°, IR (KBr) cm^−1^: 3370 (NH), 1577 (C=N), 1680 (C=0), 1526 (C=C), PMR (DMSO-d_6_) δ ppm: 2.1 (b, 2H, NH_2_), 7.0-7.9 (m, 7H, Ar-H), 8.0 (s,1H, NH), EI-MS (m/e): 355.77.SPIII-SM yield: 65%, mp: 192°, IR (KBr) cm^−1^: 3340 (NH), 1670 (C=N), 1695 (C=0), 1520 (C=C), PMR (DMSO-d_6_) δ ppm: 2.10 (s, 6H, 2 × CH_3_), 4.1 (b, 1H, -SO_2_ NH), 7.0-7.92 (m, 8H, Ar-H), 8.0 (s, 1H, NH), EI-MS (m/e): 396.42

N-acyl-isatinesulphonamide derivatives were synthesized by refluxing an equimolar mixture of (0.01 mol) isatins (5-chloro-1-acetyl-isatin, 5-bromo-1-acetyl-isatin, 5-bromo-1-benzoyl-isatin and 5-methyl-1-benzoyl-isatin) and sulphadimidine for 6 h in 10 ml of glacial acetic acid. The mixture was cooled to room temperature and poured into crushed ice; the solid thus obtained was recrystallized from ethanol. SPIII-5Br-AC yield: 67%, mp: 221°, IR (KBr) cm^−1^: 3310 (NH), 1690 (C=N), 1705 (C=0), 1510 (C=C), PMR (DMSO-d_6_) δ ppm: 2.30 (s, 6H, 2xCH_3_), 2.42 (s, 3H, CH_3_), 4.1 (b, 1H, -SO_2_ NH), 6.1 (d, 2H, pyrimidinyl), 7.0-7.92 (m, 7H, Ar-H), EI-MS (m/e):528.38. SPIII-5Br-BZ yield: 72%, mp: 285°, IR (KBr) cm^−1^: 3320 (NH), 1695 (C=N), 1707 (C=0), 1520 (C=C), PMR (DMSO-d_6_) δ ppm: 2.32 (s, 6H, 2xCH_3_), 4.1 (b, 1H, -SO_2_ NH), 6.1 (d, 2H, pyrimidinyl), 7.1-8.0 (m, 11H, Ar-H), EI-MS (m/e):590.04. SPIII-5Me-AC yield: 72%, mp: 228°, IR (KBr) cm^−1^: 3360 (NH), 1685 (C=N), 1705 (C=0), 1530 (C=C), PMR (DMSO-d_6_) δ ppm: 2.10 (s, 6H, CH_3_), 2.32 (s, 3H, CH_3_), 2.45 (s, 3H, CH_3_), 4.1 (b, 1H, -SO_2_ NH), 6.1 (d, 2H, pyrimidinyl), 7.0-7.92 (m, 7H, Ar-H), 8.0 (s, 1H, NH), EI-MS (m/e): 463.51. SPIII-5Cl-BZ yield: 83%, mp: 179°, IR (KBr) cm^−1^: 3335 (NH), 1662 (C=N), 1710 (C=0), 1530 (C=C), PMR (DMSO-d_6_) δ ppm: 2.35 (s, 6H, CH_3_), 4.1 (b, 1H, -SO_2_ NH), 6.1 (d, 2H, pyrimidinyl), 7.1-8.0 (m, 12H, Ar-H), EI-MS (m/e): 446.02

The compounds were tested for antiHIV activity against the replication of HIV-1 (III_B_) in MT-4 cells^11^. The cells were grown and maintained in RPMI 1640 medium supplemented with 10% heat-inactivated Fetal Calf Serum (FCS), 2 mM-glutamine, 0.1% sodium bicarbonate and 20 μg/ml gentamicin (culture medium). HIV-1 (HTLV-IIIB/LAI) was used in all experiments. The virus strains were propagated in MT-4 cells. Titer of virus stock was determined in MT-4 cells and the virus stock was stored at -70° until used. The inhibitory effects of the compounds on HIV-1 replication were monitored by inhibition of virus-induced cytopathic effect in MT-4 cells and were estimated by the MTT method. Briefly, 50 μl of HIV-1 (100-300 CCID_50_) were added to a flat-bottomed microtiter tray with 50 μl of medium containing various concentrations of the test compounds. MT-4 cells were added at a final concentration of 6×10^5^ cells/ml. After 5 d of incubation, at 37° the numbers of viable cells were determined by the 3-(4,5-dimethylthiazol-2-yl)-2,5-diphenyltetrazolium bromide (MTT) method. Cytotoxicity of the compounds for mock-infected MT-4 cells was also assessed by the MTT method.

To determine the susceptibility of the HIV-1 integrase enzyme towards different compounds we optimized enzyme-linked immunosorbent assays. These assays use an oligonucleotide substrate of which one oligo (5'-ACTGCTAGAGATTTT CCACACTGACTAAAAGGGTC-3') is labeled with biotin on the 3' end and the other oligo is labeled with digoxigenin at the 5' end. For the overall integration assay the second 5'-digoxigenin labeled oligo is (5'-GACCCTTTTAGTC AGTGTGGAAAATCTCTAGCAGT-3'). For the Strand Transfer assay the second oligo is missing the GT oligonucleotides at the 3'. The integrase enzyme was diluted to the same specific activity in 750 mM NaCl, 10 mM Tris pH 7.6, 10% glycerol and 1 mM β-mercaptoethanol. To perform the reaction 4 μl diluted integrase (corresponds to a concentration of WT integrase of 1.6 μM) and 4 μl annealed oligos (7 nM) was added in a final reaction volume of 40 μl containing 10 mM MgCl_2_, 5mM DTT, 20 mM HEPES pH 7.5, 5% PEG and 15% DMSO. The reaction was carried out for 1h at 37°. These reactions were followed by an immunosorbent assay on avidin coated plates^13^

**TABLE 2:** INHIBITION OF HIV-1 INTEGRASE ACTIVITY

Compounds	IC_50_^a^ (μM/ml)	IC_50_^b^ (μM/ml)
SPIII-5Cl-AC	>250	>250
SPIII-5Me-AC	53.62±11.67	69.22±1.68
SPIIII-5Br-AC	>250	>250
SPIII-5Br-BZ	>250	>250
Pyranodipyrimidines (STD)	0.03±0.01	0.09±0.03

a 50% inhibitory concentration or concentration of the compound required to inhibit the overall integration reaction by 50%

b 50% inhibitory concentration or concentration of the compound required to inhibit the strand transfer reaction by 50%. All the data represent mean value±SD for at least two separate experiments

4-(1-acetyl-5-methyl-2-oxoindolin-3-ylideneamino)-N-(4,6-dimethylpyrimidin-2-yl)benzene sulfonamide (SPIII-5ME-AC) inhibits the HIV Integrase enzymatic activity and 4-(1-benzoyl-5-chloro-2-oxoindolin-3-ylideneamino)-N-(4,6-dimethylpyrimidin-2-yl)benzenesulfonamide (SPIII-5Cl-BZ) exhibits 36 percent maximum protection against HIV-1 at subtoxic concentration. None of the compounds exhibited antiHIV effect and all the compounds displayed cytotoxic properties in the lymphocyte cell line (MT-4 cells). The 50% effective concentration (EC_50_) values of the synthesized compounds against the replication of HIV-1(IIIB) in acutely infected MT-4 cells were higher than the cytotoxic concentration (CC_50_). Lead molecules isatins (isatin, 5-chloroisatin, 5-bromoisatin, and 5-methylisatin) and sulphadimidine not active against HIV-1(IIIB) in MT-4 cells, but the combined product isatinesulphadimidine (SPIII, fig. 1 and Table 1) were active against HIV-1 and 2 in MT-4 cells^13^. Presence of 4,6-dimethylpyrimidinyl group in SO_2_NH_2_ of Isatine-sulphadimidine (SPIII) lead molecule is essential for antiHIV activity. Substitution in NH group of isatin (N-acylation) abolishes the antiHIV activity of the lead molecule SPIII (Table 1).

**Fig. 1:**
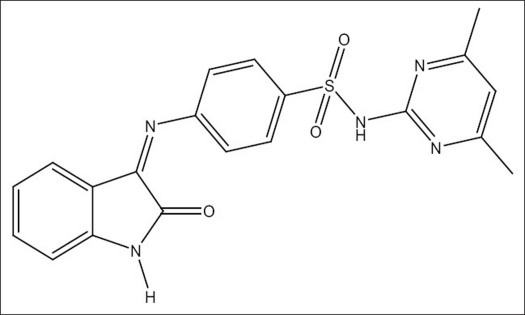
Structure of SPIII lead molecule

**TABLE 1:** ANTIHIV ACTIVITY AND CYTOTOXICITY OF ISATIN IN MT-4 CELLS

Compounds	EC_50_^a^ (μg/ml)	CC_50_^b^ (μg/ml)	Max Protection
IS	>42.2	42.2	1
5Br IS	>9.4	9.4	2
5Cl IS	>29.5	29.5	2
5F IS	>8.5	8.5	5
5Me IS	>50	>50	6
SD	>73.02	73.02	1
SPIII	8	>125	148@
SPIII-SM	>125	>125	8
SPIII-S	>50	>50	4
SPIII-SMe	>50	>50	8
SPIII-SCl	>13.6	13.6	4
SPIII-5Br-AC	>101.68	101.68	1
SPIII-5Br-BZ	>86.23	86.23	1
SPIII-5Me-AC	>116.06	116.06	3
SPIII-5Cl-BZ	> 56.47	56.47	36
AZT	0.0064	65.06	106

a Concentrations of each compound required to inhibit the CPE of retroviruses in MT-4 cells by 50%.

b Concentrations required to cause cytotoxicity to 50% of the MT-4 cells. @ SPIII lead value was taken from references 11 and 12
